# Antibacterial soap use impacts skin microbial communities in rural Madagascar

**DOI:** 10.1371/journal.pone.0199899

**Published:** 2018-08-20

**Authors:** James J. Yu, Melissa B. Manus, Olaf Mueller, Sarah C. Windsor, Julie E. Horvath, Charles L. Nunn

**Affiliations:** 1 Department of Evolutionary Anthropology, Duke University, Durham, North Carolina, United States of America; 2 Duke Global Health Institute, Durham, North Carolina, United States of America; 3 Department of Molecular Genetics and Microbiology, Duke University, Durham, North Carolina, United States of America; 4 Center for the Genomics of Microbial Systems, Duke University School of Medicine, Durham, North Carolina, United States of America; 5 Genomics & Microbiology Research Lab, North Carolina Museum of Natural Sciences, Raleigh, North Carolina, United States of America; 6 Department of Biological and Biomedical Sciences, North Carolina Central University, Durham, North Carolina, United States of America; Argonne National Laboratory, UNITED STATES

## Abstract

The skin harbors diverse communities of microorganisms, and alterations to these communities can impact the effectiveness of the skin as a barrier to infectious organisms or injury. As the global availability and adoption of antibacterial products increases, it is important to understand how these products affect skin microbial communities of people living in rural areas of developing countries, where risks of infection and injury often differ from urban populations in developed countries. We investigated the effect of antibacterial soap on skin microbial communities in a rural Malagasy population that practices subsistence agriculture in the absence of electricity and running water. We quantified the amount of soap used by each participant and obtained skin swab samples at three time points: prior to soap use, immediately after one week of soap use, and two weeks after soap use was discontinued. Soap use did not significantly impact ecological measures of diversity and richness (alpha diversity). However, the amount of soap used was a predictor of community-level change (beta diversity), with changes persisting for at least two weeks after subjects stopped using soap. Our results indicate that the overall species richness of skin microbial communities may be resistant to short-term use of antibacterial soap in settings characterized by regular contact with the natural environment, yet these communities may undergo shifts in microbial composition. Lifestyle changes associated with the use of antibacterial soap may therefore cause rapid alterations in skin microbial communities, with the potential for effects on skin health.

## Introduction

The microbes (bacteria, fungi, protozoa, Archaea) that live in and on the human body are abundant and diverse, with important implications for health [1–4]. These microbes are thought to have co-evolved with humans in relation to the body sites they inhabit, providing an array of functions for the host, ranging from digestion to immune defense [5]. While research endeavors such as the Human Microbiome Project [6] aim to establish the normal range of variation in human microbiomes (encompassing all microbes, their genes, and functions) and effects on health, this task has been challenging due to the marked variation of microbial communities (groups of microorganisms that coexist in a mutual space) within and across populations [7]. With the exception of a few studies [8–10], microbiome research has focused on people living in Western settings, such as the United States and Europe, and is predominantly centered around the gut microbiome.

Less is known about the microbial communities on human skin, despite it being the largest organ and home to a variety of bacteria and other organisms important for health [11]. The skin also acts as an interface between the human body and the outside environment, and skin anatomy and physiology allow different microbes to flourish across various skin sites [12]. While most skin communities are characterized by four bacterial phyla (Actinobacteria, Firmicutes, Bacteroidetes, and Proteobacteria), the species-level diversity of skin microbes is much more robust [12]. Due to the dynamic nature of the skin microbiome and the role of the skin as an intermediary between humans and the outside world, efforts have been made to understand the development of skin microbial communities [13]. These studies reveal that immediately after birth, infant skin microbial communities are relatively homogenous across body sites [14]. Colonization of the skin with new microbial organisms does not begin until about three months of age, and bacterial composition at a young age may have an impact on an individual’s microbiome and health in the future, possibly affecting the skin microbiome’s stability and immune function [14]. However, these bacterial communities change over time, with body sites eventually differing in microbial composition as a child matures [13]. For example, physiological changes throughout puberty, such as increased density and thickness of body hair, drastically impact the overall structure of skin microbial communities by altering the local ecosystem of certain skin sites and the microbial communities that each site can support [13].

In addition to changes that occur throughout development, human skin bacterial communities also undergo alteration due to environmental variables. The body can be viewed as an island that is in constant interaction with different microbes over time, with a wide variety of environmental and biological factors influencing the diversity and abundance of these microorganisms [11, 15]. In the case of both skin and gut microbial communities, certain resident bacteria are able to slow or even resist the invasion of other taxa [15–18]. This can lead to long-term stability of microbial communities, with disruption occurring only when these communities are significantly perturbed [15, 16]. Environmental characteristics of the skin also affect bacterial composition [19]. For example, variation in physiological traits make some skin sites more conducive to bacterial survival and proliferation [20], with moist or sebaceous skin sites (such as the armpit or inner elbow) tending to harbor a higher abundance and diversity of bacteria than dry skin sites (inner forearm or buttock) [21, 22]. Skin microbial communities also vary across human populations, due to differences in host genetics, cultural practices, and geography [9, 10]. As examples, one study concluded that the skin microbiomes of individuals from China differ greatly from that of Westerners–the genus *Enhydrobacter* was found to be common on the skin of the Chinese participants but not on the skin of Westerners [10]. Another study found striking differences when comparing Amerindians of South America to populations in New York and Colorado, with the United States individuals’ forearms dominated by *Propionibacterium*, one of the groups of Amerindians’ forearms dominate by *Staphylococcus*, and the other group of Amerindians’ forearms significantly more diverse than the other two groups [9]. These differences have been attributed to lifestyle heterogeneity that results in variation in environmental exposures–including exposures to soil, water, and plants–and the intensity of these exposures, where greater amounts of contact with the outside environment may result in a stronger environmental signature.

Behavioral practices can also impact skin microbial communities [11, 12, 21, 23, 24]. Handwashing removes dirt, organic material, and microorganisms, with different techniques resulting in varying degrees of “cleanliness” [25]. For example, duration and friction play a key role in handwashing; the Association for Professionals in Infection Control and Epidemiology (APIC) recommends that people, “vigorously rub hands together for 10 to 15 seconds.” Other behavioral differences that impact handwashing effectiveness include glove use, nail polish, jewelry, types of hand lotion used, and compliance to standards set by the workplace. Additionally, while plain soap works by binding to dirt and organic material, antiseptic soaps contain specific bactericidal active ingredients that eliminate microorganisms, both pathogenic and beneficial [23].

While people in developed countries often have access to antibacterial products and are encouraged to maintain high levels of personal hygiene through public health programs and cultural norms, these products are much less common in low and middle-income countries (LMICs). As some LMICs transition toward increased development, access to and use of antibacterial soaps may increase. In addition, as LMICs see an increase in antibiotic resistance, the addition of more antibiotic products, including soap, is of concern. The net benefits of antibacterial soap on human health is unclear and potentially even harmful, as evidenced by the US Food and Drug Administration’s (FDA) recent ruling to prohibit the sale of soaps and shampoos containing certain antibacterial ingredients [26]. While some studies highlight ingredients in antibacterial soap that can be harmful to human and environmental health, more research is needed to determine the effects of antibacterial soap use across a range of diverse human populations [27–32]. Our study is intended to expand our understanding of the effects of antibacterial soap use on humans in a non-Western setting.

We aimed to assess the impact of antibacterial soap on skin bacterial communities in a population in rural Madagascar. This population is valuable in this context for several reasons. First, subsistence agricultural is commonly practiced without powered machinery, putting people in close contact with elements of the natural environment, including domesticated and wild animals. Due to this increased diversity and intensity of environmental exposures, the effects of antibacterial soap on skin microbial communities may differ from effects in Western populations. Second, this population lacks access to running water, and instead uses naturally flowing, mountain-derived water for bathing. Use of this common water source, which contrasts with Western plumbing and bathing practices, may homogenize microbial communities across individuals. Third, this rural community lacks access to Western hygiene products, including antibacterial soap. Hence, skin bacterial communities may be less disturbed on average, providing context for understanding how antibacterial soap alters microbial communities in novel ways. Finally, this population is increasingly exposed to aspects of the Western lifestyle through economic development. Moving forward, demand for Western products, including soap, is likely to increase, making it important to investigate how the use of antibacterial soaps affects skin microbial communities, and in turn, health.

We investigated the hypothesis that antibacterial soap use impacts skin microbial communities in this rural population. By comparing an experimental group with access to antibacterial soap to a control group without access to antibacterial soap, we tested the following predictions:

1. Individuals using antibacterial soap exhibit a greater change in taxonomic richness (alpha diversity) after one week of soap use, as compared to a matched control group that was not given access to antibacterial soap.
2. Change in alpha diversity of microbial taxa covaries with the amount of soap used, consistent with a dose-response relationship.
3. Individuals using antibacterial soap exhibit a greater change in taxonomic richness after having stopped soap use for two weeks, compared to individuals that did not use antibacterial soap over the same time period.
4. Individuals using antibacterial soap exhibit a greater change in the composition of skin microbial communities across sampling periods (beta diversity).
5. Changes in beta diversity covary with amount of antibacterial soap used, consistent with a dose-response relationship.
6. Because the composition of the microbial community may inhibit the invasion of new microorganisms, changes in community composition following antibacterial soap use would persist for at least two weeks after discontinuing soap use.

## Materials and methods

### Participants and experimental design

The study was conducted in Mandena, Madagascar (approximately -18°42'00” S 47°50'00” E), located in the SAVA region (an acronym for the four major cities in the region: Sambava, Antahala, Vohémar, and Andapa). The village consists of approximately 3,000 people and is adjacent to Marojejy National Park. We first recruited individuals at a village-wide meeting, where the project was introduced and interested individuals were invited to participate in the study on future dates. Upon their return to our health clinic and after obtaining informed written consent through Malagasy translators, we obtained basic health measurements for each participant, including temperature, blood pressure, heart rate, height, and weight. Participants with clinically elevated health measurements, open wounds, infirmity, or illness were excluded from enrolling in the study. Of the eligible participants, we enrolled 20 adult males that ranged in age from 18 to 75 years. After obtaining a second informed written consent facilitated by Malagasy translators, ten individuals were randomly assigned to the experimental group to use antibacterial soap for one week, while the other ten individuals were assigned to the control group that would not be administered antibacterial soap. We selected individuals at random because most participants reported using only local soap, which we observed was used for bathing, washing clothes, and cleaning dishes. We administered Santex antibacterial soap (containing Triclocarban, an antimicrobial agent common in Western hygiene products) [33] to the experimental group, as it was difficult to acquire in Mandena and would only be used by the experimental group with explicit instructions to solely use the soap for bathing. At the conclusion of the study, all subjects were given two bars of Santex soap as compensation for participation. Surveys that inquired about basic health, lifestyle, and demographic characteristics were administered with the help of local Malagasy translators. All surveys and sample collection were approved by the Duke University Institutional Review Board (IRB Protocol C0848) and local Malagasy health authorities.

We sampled each individual at four skin sites (right ankle, right medial forearm, right outer hand, right armpit) using sterile, dual-tipped rayon swabs (Fisher BD BBL CultureSwab, Media-free). All skin swab samples were obtained by rubbing the swab across the site for 30 seconds, with all sampling conducted by authors MBM and JJY. We provided individuals in the experimental group with both written and verbal instructions to maintain their typical bathing schedule, but to use the soap on specific body parts at each bath time (i.e., the four body sites that we sampled) and to refrain from sharing the soap with family members. We did not provide the control group with instructions regarding soap use because avoiding hygienic practices in this rural environment could put these individuals at risk of disease, and we wished to minimize disruptions to the typical behaviors of our participants. All subjects were swabbed at three different time points to investigate how antibacterial soap use impacts skin microbial composition. The first sampling event, defined as Time Period 1, took place before the introduction of soap (to obtain a baseline skin microbial community sample); the second, Time Period 2, occurred after one week (7–8 days) of antibacterial soap use (to investigate the immediate effects of soap use); and the third, Time Period 3, occurred two weeks (14–15 days) after discontinuation of antibacterial soap use (to determine any lasting effects of antibacterial soap use) (Table 1). We collected the remaining soap at Time Period 2 to ensure discontinuation of antibacterial soap use in the experimental group. At each sampling event, all participants were swabbed once over a span of three days, regardless of whether they received soap. The soap that was administered to the experimental group was weighed at the beginning and end of Time Period 2 to quantify the amount of soap used by each individual. One participant used his entire bar of soap before the sampling event at Time Period 2 and was given a second bar.

**Table 1:** Time periods of sampling time of both experimental group and control group.

Time Period	Event
1	Before introduction of soap to experimental group (to obtain a baseline skin microbial community sample)
2	After one week (7–8 days) of antibacterial soap use in experimental group (to investigate the immediate effects of antibacterial soap use)
3	Two weeks (14–15 days) after discontinuation of antibacterial soap use in experimental group (to determine any lasting effects of antibacterial soap use)

The swab samples were stored in a refrigerator powered by a generator when available, or inside a plastic cooler at room temperature with a daytime mean of 20°C (David Samson, personal communication, 2017). Because Mandena is not supported by electricity and a laboratory was inaccessible, we stored the samples in a uniformly cool environment in the field and during transit, rather than exposing samples to repeated freeze-thaw cycles. A previous study found that differences in short-term storage conditions did not significantly alter microbial communities, and that samples stored in non-freezing conditions were still useful for analyses [34]. Samples were transported to the United States on three separate flights (all within two weeks of collection), using insulated envelopes with ice packs on the plane and hotel room refrigerators when possible during layovers. Upon arrival in the United States, they were stored at -80°C at the North Carolina Museum of Natural Sciences in Raleigh, NC until the DNA extraction phase.

### Lab procedures

DNA extraction followed the MO BIO Powersoil DNA Isolation Kit (MO BIO, before merging with Qiagen) protocol with two modifications (S1 Text). The extracted DNA was then sent to the Duke University Sequencing Core for library preparation and sequencing of the 16S rRNA V3-V4 region (S2 Text).

### Data analysis

44,308,999 300 bp MiSeq read pairs were joined in QIIME (Quantitative Insights into Microbial Ecology, 1.9.1), with join_paired_ends.py, using the fastq-join method with a maximum percentage difference (-p) of 25%. Assembled amplicons were further quality filtered with split_libraries_fastq.py (phred quality Q20), resulting in 20,583,303 sequences used for downstream analysis. 16S rRNA OTUs were picked using the closed reference method with SortMeRNA and greengenes database (version 13.8) at 97% similarity. A minimal sampling depth (-e) of 8,000, and otherwise default parameters, were used for subsequent diversity analyses with core_diversity_analyses.py of the QIIME package. All QIIME pipeline scripts were run on the Duke Compute Cluster. All sequence reads have been deposited at GenBank under the accession KBXC00000000.

To ensure there were no biases between the experimental group and control group, we were first compared the two groups with two separate t-tests, one comparing Shannon diversity index between the two groups and the other comparing observed OTUs between the two groups. We found no significant differences between the two groups for either measure of alpha diversity. To investigate the impact of antibacterial soap on alpha diversity of skin microbial communities, we compared the change in bacterial taxa for all individuals between Time Period 1 and 2, grouping together individuals that used soap (experimental group) and individuals that did not use soap (control group). We quantified alpha diversity as taxonomic richness (number of OTUs), and as combined taxonomic richness and abundance (Shannon diversity index). We used paired t-tests with an alpha of 0.05 for observed OTUs and Shannon diversity indices for both the experimental and control groups, comparing alpha diversity in Time Period 1 and 2 matched by subject (Prediction 1). We repeated this to compare between Time Period 2 and 3, following discontinuation of soap use. To investigate if change in alpha diversity covaries with amount of soap used (Prediction 2), we used linear regression models including both the control and experimental group to assess whether the amount of antibacterial soap used had an impact on the number of OTUs or the Shannon diversity index from Time Period 1 to 2. We repeated this process from Time Period 2 to 3 to investigate the relationship between discontinuing soap use and resulting alpha diversity (Prediction 3). We included amount of soap used and subject’s age as predictors of change in alpha diversity.

We defined beta diversity as the UniFrac distance between two of the same samples (collected from the same body site on the same individual) from different time periods [35]. A greater UniFrac distance indicates greater dissimilarity between two samples, while a smaller change indicates more similarity. To investigate the impact of antibacterial soap on beta diversity (Prediction 4), we combined all the samples from both the experimental and control group, and then created a general linear regression model to predict the UniFrac distance between two of the same samples from Time Period 1 and Time Period 2, using amount of soap used and age as predictors (to determine a dose-response relationship, Prediction 5). To test whether antibacterial soap use had an impact on microbial composition two weeks after discontinuation of soap (Prediction 6), we generated a general linear mixed model including both the experimental group and control group to predict beta diversity with time period, amount of soap used, and age as fixed effects and participant identity as a random effect. All statistical tests were run in R version 3.1.2 [36]. We used linear discriminant analysis effect size (LEfSe) analysis for the experimental group, separated by time period, to investigate differential abundance of particular microbial taxa affected by soap use [37].

## Results

**Fig 1:**
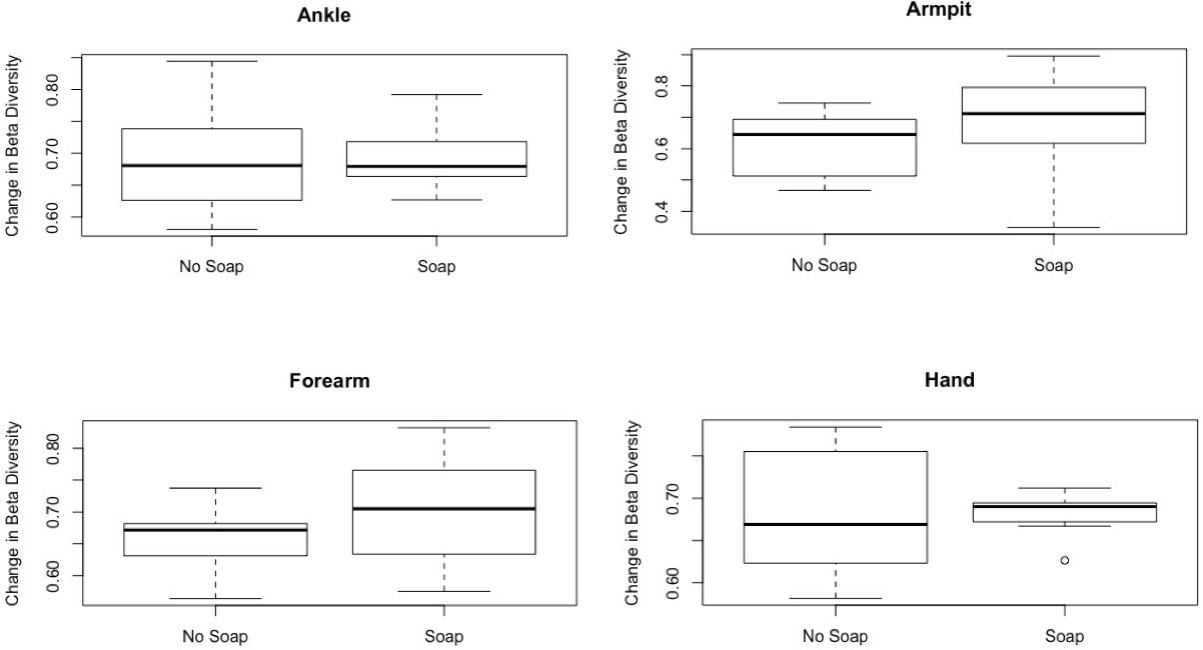
Comparison of change in beta diversity for experimental group versus control group differentiated by skin site.

**Fig 2:**
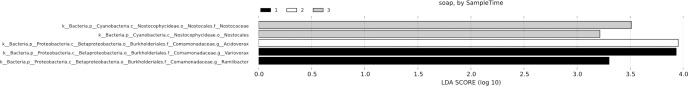
Top discriminative taxa determined by LEfSe analysis for different time periods for the individuals that received soap.

The amount of soap used significantly predicted beta diversity from Time Period 1 to Time Period 2 (Table 2). The coefficient indicated a positive dose-response relationship, i.e., more soap use yielded greater beta diversity. This variable also predicted beta diversity in a general linear mixed model, although sampling period did not (Table 3). At all four body sites, with the exception of ankles, soap use was associated with an increase in beta diversity from Time Period 1 to Time Period 2 (Fig 1). LefSe analysis results showed that for the experimental group, differences in microbial communities were at least partially driven by the genera *Ramlibacter* and *Variovorax* for Time Period 1 (before soap use) and *Acidovorax* for Time Period 2 (after one week of soap use) (Fig 2).

**Table 2:** General linear regression model predicting change in beta diversity from Time Period 1 to 2 for all subjects.

Predictor	Coefficient	p-value	t-value	Std. error
Soap Used	0.00146	0.0261*	2.78	0.000642
Age	0.00131	0.0865	1.74	0.000757
Armpit	-0.0347	0.238	-1.19	0.0291
Forearm	-0.0101	0.740	-0.333	0.0303
Hand	-0.00784	0.793	-0.263	0.0298

Note: The body site predictor uses the ankle as the reference category.

Significant p-values are marked with an asterisk (*).

d.f. = 70.

**Table 3:** General linear mixed model predicting change in beta diversity with sampling period, amount of soap used, and age as predictors, and participant as a random effect.

Predictor	Coefficient	t-value	Std. error
Sampling Time	0.00400	0.236	0.0170
Soap Used	0.00180	3.47*	0.000519
Age	0.000982	1.56	0.000630

**Note:** Significant t-values are marked with an asterisk (*).

We found no significant differences in alpha diversity (number of OTUs or Shannon diversity index) between samples collected at Time Periods 1 and 2 (Table 4). The control group showed a significant difference in Shannon diversity between Time Periods 2 and 3, while the difference in the number of OTUs approached significance (Table 5). In contrast, neither number of OTUs nor Shannon diversity differed significantly between Time Periods 2 and 3 for the experimental group (Table 5). The amount of soap used or body site was not a significant predictor of change in these metrics across the three time periods.

**Table 4:** Variation in OTUs and Shannon diversity index from Time Period 1 to Time Period 2.

Measurement	Group	Mean difference	t-statistic	d.f.	p-value
Number of OTUs	Control	-84.21	-1.646	34	0.109
Number of OTUs	Experimental	2.68	0.040	31	0.968
Shannon diversity index	Control	-0.2363	-1.129	34	0.267
Shannon diversity index	Experimental	-0.4042	-1.502	31	0.143

**Note:** Mean difference is calculated as Time Period 2 –Time Period 1, such that negative differences reflect a decline in the metric during the soap use period. The control group was not given access to soap, while the experimental group was given access to soap.

**Table 5:** Variation in OTUs and Shannon diversity index from Time Period 2 to Time Period 3.

Measurement	Group	Mean difference	t-statistic	d.f.	p-value
Number of OTUs	Control	108.64	1.983	30	0.057
Number of OTUs	Experimental	4.4281	0.065	31	0.949
Shannon diversity index	Control	0.6072	2.526	30	0.017*
Shannon diversity index	Experimental	0.4421	1.312	31	0.199

**Note:** Mean difference is calculated as Time Period 3 –Time Period 2. The experimental group discontinued soap use for 2 weeks from Time Period 2 to Time Period 3.

Significant p-values are marked with an asterisk (*).

## Discussion

We found that soap use impacts the community composition of microbes on human skin, but may not affect the taxonomic richness of these communities. Changes in community composition persisted for at least two weeks in the experimental group, which suggests that continued use of antibacterial soap may have long-term effects on skin microbial communities. Similar effects have been documented for the use of other hygienic products, such as antiperspirant and deodorant [38].

Although we expected that use of antibacterial soap would change the taxonomic richness of skin microbes (Prediction 1), we failed to find any evidence of that relationship, as measured by the number of OTUs and Shannon diversity index across the four body sites (Table 4). Moreover, there was no effect across Time Period 1 to 2 (soap use) or Time Period 2 to 3 (post-soap use) (Prediction 3). It could be that our small sample size–associated with the challenges of fieldwork in a remote location–limited our ability to detect significant effects. However, the direction of statistically significant results in additional analyses was opposite to that of our predictions, suggesting there was sufficient power in our sample size to detect differences in taxonomic richness. In addition, each of the 20 participants contributed samples from four different body sites over three time periods, thus vastly increasing our sample size while controlling for the individual sampled.

Despite the lack of change in alpha diversity, we found support for predictions related to changes in beta diversity. There was greater dissimilarity in microbial communities across time in samples from the experimental group than in samples from the control group (Table 2, Prediction 4). This indicates soap use is a driver of variation in microbial community composition. In addition, the magnitude of the coefficients in our models covaried with the amount of soap used (Table 2), indicating a dose-response relationship (Table 2, Prediction 5). Interestingly, soap use covaried with greater beta diversity (i.e., larger UniFrac distances) for all skin sites except for the ankle (Fig 1). This may be due to increased contact between ankles and the outside environment, compared to other body sites. This is in line with findings from a similar study in Mandena, where ankle samples exhibited greater similarity to domesticated cattle than did other body sites, likely due to shared environment of unshod human feet and cattle while working in the agricultural fields [8]. In this light, perhaps soap use affected microbial communities on body sites that contained typical human skin microbes, whereas ankles contained more environmentally-sourced microbes that were less affected by soap use.

Our LefSe analysis (Fig 2) indicated that the differences in microbial communities over the three time periods were driven by the genera *Ramlibacter*, *Variovorax*, and *Acidovorax*, all of which are part of the family *Comamonadaceae*, which consists of environmental bacteria from water and soil, as well as certain pathogens [39]. It may be that soap use reduced certain bacterial taxa, allowing other formerly unsuccessful taxa to colonize the open niches. Previous research demonstrated that bacteria compete for limited resources, and that when certain bacterial strains die, other species can successfully colonize the area [15, 19]. While this would present a community-level shift in composition, it may result in minimal changes in overall species richness (i.e., a one-to-one species turnover). This could also explain our finding that soap use does not predict change in alpha diversity, but does have an effect on beta diversity. In this rural setting, individuals come into close contact with multiple, diverse sources of environmental bacteria [8]. This could result in two, non-mutually exclusive phenomena: (i) after soap use, open niches on the skin could quickly be filled by environmentally-derived taxa that normally are unable to compete with the resident skin microorganisms; and (ii) irrespective of soap use, human skin microbial communities harbor taxa that are typically considered to be environmentally sourced. The latter could explain the unexpected finding that Shannon diversity index significantly increased in samples from the control group (Table 5). In addition, it may be that the introduction of soap prompted the experimental group to bathe more frequently than is typical in this setting (and perhaps more frequently than the control group), due to an induced pressure to use the soap and present clean skin at sample collection events. The common practice of bathing outdoors in the communal river could lead to increased exposure to water and soil taxa, therefore counteracting effects of soap use on skin microbial communities that are observed in Western settings.

Importantly, time period was not a significant predictor of beta diversity based on our general linear mixed model (Table 3), indicating that shifts in microbial communities persisted for at least two weeks (Prediction 6). This is relevant for considering potential long-term health impacts of antibacterial soap. Further studies could investigate the duration of the effects of soap, and whether they can induce a permanent change in microbial community composition. This effect on microbial communities may also be an avenue for the emergence of antimicrobial resistance; as such, future studies should target bacterial taxa that are most likely to acquire resistance. The World Health Organization (WHO) and Centers for Disease Control and Prevention (CDC) have acknowledged antimicrobial resistance as a priority for public health, making this study relevant to health concerns globally [40, 41].

In 2013, the FDA urged continued research on the possible effects of antibacterial soap use, especially soaps containing the active ingredients Triclosan and Triclocarban. Three years later, the FDA prohibited the sale of hygiene products containing these antibacterial agents [26]. As the soap used in our study contained Triclocarban, these results are relevant to concerns raised by governmental organizations and citizen advisory groups, and future studies should continue to investigate the effects of these products on health. While evidence suggests that soap use is more effective at reducing bacterial contamination than is washing with only water [23], recent studies indicate that washing with antibacterial soap is no more effective at reducing bacterial levels than is the use of regular soap [42–45]. Similarly, antibacterial soap is no more effective at reducing disease incidence on a community level than is non-antibacterial soap [24]. In light of these studies, more work is needed to disentangle health outcomes related to the use of these products.

Other studies have identified a wide variety of associated environmental health concerns from antibacterial product use [31]. These effects include contamination of public water sources with antimicrobial agents [27, 32] and pathogenic effects on the gut microbial communities of rats [28, 29]. Because people in Mandena predominantly wash their bodies, clothes, and dishes (regardless of soap use) in a communal river, there is concern that negative side effects of human antimicrobial product use could affect the environment and non-human animals [46]. It is possible that frequent contact with domesticated animals (many of which are vitally important to livelihoods in Madagascar) facilitates transfer of antimicrobial chemicals that persist on human skin after using these products. For example, results from a similar study conducted at this site [8] suggest that if chemical properties of antimicrobial products persist on human skin or in the environment, they could be transferred to domesticated cattle that come into frequent contact with humans.

There is a growing interest in the microbiomes of non-Western populations [8–10, 47], and some studies have found that these populations harbor greater microbial diversity than do Western populations [9, 47]. Our results indicated that while antibacterial soap may not directly affect microbial taxonomic richness, it may lead to changes in the composition of skin microbial communities. This highlights the importance of understanding the impacts of soap on microbial communities of individuals without previous exposure to these products, especially as globalization is likely to increase the use of antimicrobials in non-Western settings. This is relevant to theories such as the Old Friends Hypothesis [48, 49] and the Biome Depletion Hypothesis [50], which suggest that a reduction in microbial diversity may result in detrimental immune system development. In this light, future work in settings like Mandena should investigate whether individuals do in fact harbor beneficial microbes and the consequences of losing them, particularly in settings that are targeted for global public health initiatives that promote soap use.

In conclusion, we found that antibacterial soap impacts the structure of microbial communities, and that these changes persist for at least two weeks. These findings suggest that antibacterial products have a lasting impact on skin microbes, and are especially important in the context of changing lifestyles throughout the developing world, with increases in wealth likely to be associated with increased demand for hygienic products such as antibacterial soaps. While the efficacy of antibacterial soap and bactericidal products has been under scrutiny, the health-related effects of these products on skin microbial communities has yet to be examined thoroughly. Future research would benefit from considering additional environmental and behavioral factors that have the potential to impact the skin microbial communities in rural settings, such as interactions with non-human animals, occupation, and use of shoes.

## Supporting information

S1 Text DNA extraction protocol. (DOCX)

S2 Text Metagenomic library prep. (DOCX)

S1 Data Data BetaDiversity1to2. (CSV)

S2 Data Data BetaDiversity. (CSV)

S3 Data Data AlphaDiversityModel1to2. (CSV)

S4 Data Data AlphaDiversityModel2to3. (CSV)

## References

[1] Blaser MJ *Missing Microbes*: *How the Overuse of Antibiotics Is Fueling Our Modern Plagues*. New York: Henry Holt, 2014. Print. 10.1096/fj.14-0901ufm PMC4139909 29405744

[2] Knight R, & Buhler B. *Follow your gut*: *The enormous impact of tiny microbes*. New York: Ted Books, Simon & Schuster; 2015.

[3] Pflughoeft KJ and Versalovic J. "Human Microbiome in Health and Disease." *Annual Review of Pathology*: *Mechanisms of Disease* 2012;7(1): 99–122. 10.1146/annurev-pathol-011811-132421 21910623

[4] The Human Microbiome Project Consortium. Structure, function, and diversity of the healthy human microbiome. Nature. 2012;486. 10.1038/nature11234 PMC3564958 22699609

[5] Balter M. "Taking Stock of the Human Microbiome and Disease." AAAS. 2012;336(June): 1246–1247. 10.1126/science.336.6086.1246 22674333

[6] Turnbaugh PJ, Ley RE, Hamady M, Fraser-Liggett CM, Knight R, Gordon JI. "The human microbiome project: a strategy to understand the microbial components of the human genetic and metabolic landscape and how they contribute to normal physiology and predisposition to disease." *Nature*. 2007;449: 804+. 10.1038/nature06244 17943116 PMC3709439

[7] Foxman B and Goldberg D. "Why the human microbiome project should motivate epidemiologists to learn ecology." Epidemiology. 2010;21(6): 757–759. 10.1097/EDE.0b013e3181f4e1f9 20924228 PMC3715124

[8] Manus MB, Yu JJ, Park LP, Mueller O, Windsor SC, Horvath JE, Nunn CL. Environmental influences on the skin microbiome of humans and cattle in rural Madagascar. *Evolution*, *Medicine*, *and Public Health*, 2017; eox013, 10.1093/emph/eox013 PMC5631097 29147568

[9] Blaser MJ, Dominguez-Bello MG, Contreras M, Magris M, Hidalgo G, Estrada I, et al "Distinct cutaneous bacterial assemblages in a sampling of South American Amerindians and US residents." ISME J. 2013;7(1): 85–95. 10.1038/ismej.2012.81 22895161 PMC3526177

[10] Leung MHY, Wilkins D, Lee PKH. "Insights into the pan-microbiome: skin microbial communities of Chinese individuals differ from other racial groups." Sci Rep. 2015;5: 11845 10.1038/srep11845 26177982 PMC4503953

[11] Rosenthal M, Goldberg D, Aiello A, Larson E, Foxman B. "Skin microbiota: microbial community structure and its potential association with health and disease." *Infect Genet Evol*. 2011;11(5): 839–848. 10.1016/j.meegid.2011.03.022 21463709 PMC3114449

[12] Grice EA and Segre JA. "The skin microbiome." Nature Reviews Microbiology. 2011;9: 244+. 10.1038/nrmicro2537 21407241 PMC3535073

[13] Oh J, Conlan S, Polley EC, Segre JA, Kong HH. "Shifts in human skin and nares microbiota of healthy children and adults." *Genome Med*. 2012;4(10): 77 10.1186/gm378 23050952 PMC3580446

[14] Capone KA, Dowd SE, Stamatas GN, Nikolovski J. "Diversity of the human skin microbiome early in life." J Invest Dermatol. 2011;131(10): 2026–2032. 10.1038/jid.2011.168 21697884 PMC3182836

[15] Costello EK, Stagaman K, Dethlefsen L, Bohannan BJ, Relman DA. "The Application of Ecological Theory Toward an Understanding of the Human Microbiome." Science Magazine. 2012;336(June): 1255–1262. 10.1126/science.1224203 PMC4208626 22674335

[16] Fierer N, Ferrenberg S, Flores GE, González A, Kueneman J, Legg T, et al "From Animalcules to an Ecosystem: Application of Ecological Concepts to the Human Microbiome." Annual Review of Ecology, Evolution, and Systematics. 2012;43(1): 137–155.

[17] Brandt LJ, Aroniadis OC, Mellow M, Kanatzar A, Kelly C, Park T, et al Long-term follow-up of colonoscopic fecal microbiota transplant for recurrent clostridium difficile infection. *The American Journal of Gastroenterology*. 2012;107(7), 1079–1087. 10.1038/ajg.2012.60 22450732

[18] Petrof EO, Gloor GB, Vanner SJ, Weese SJ, Carter D, Daigneault MC, et al Stool substitute transplant therapy for the eradication of *Clostridium difficile* infection: “RePOOPulating” the gut. *Microbiome*. 2013;1, 3 10.1186/2049-2618-1-3 10.1186/2049-2618-1-3 24467987 PMC3869191

[19] Kong HH and Segre JA. "Skin microbiome: looking back to move forward." J Invest Dermatol. 2012;132(3 Pt 2): 933–939. 22189793 10.1038/jid.2011.417 PMC3279608

[20] Grice EA, Kong HH, Conlan S, Deming CB, Davis J, Young AC, et al "Topographical and temporal diversity of the human skin microbiome." Science. 2009;324(5931): 1190–1192. 10.1126/science.1171700 19478181 PMC2805064

[21] Costello EK, Lauber CL, Hamady M, Fierer N, Gordon JI, Knight R. "Bacterial community variation in human body habitats across space and time." Science. 2009;326(5960): 1694–1697. 10.1126/science.1177486 19892944 PMC3602444

[22] Council SE, Savage AM, Urban JM, Ehlers ME, Skene JHP, Platt ML, et al Diversity and evolution of the primate skin microbiome. *Proceedings of the Royal Society B*: *Biological Sciences*. 2016;283(1822), 20152586 10.1098/rspb.2015.2586 10.1098/rspb.2015.2586 26763711 PMC4721104

[23] Burton M, Cobb E, Donachie P, Judah G, Curtis V, Schmidt WP. The Effect of Handwashing with Water or Soap on Bacterial Contamination of Hands. *International Journal of Environmental Research and Public Health*. 2011;8(1), 97–104. 10.3390/ijerph8010097 10.3390/ijerph8010097 21318017 PMC3037063

[24] Luby S, Agboatwalla M, Feikin D, Painter J, Billhimer W, Altaf A, et al Effect of handwashing on child health: a randomised controlled trial. *The Lancet*. 2005;366(9481), 225–233. 10.1016/s0140-6736(05)66912-7 16023513

[25] Larson EL. APIC guidelines for handwashing and hand antisepsis in health care settings. American Journal of Infection Control. 1995; 10.1016/0196-6553(95)90070-5. 7503437

[26] FDA. FDA Issues Final Rule on Safety and Effectiveness of Antibacterial Soaps. U.S. Department of Health and Human Services 13 11 2016 Available from: http://www.fda.gov/NewsEvents/Newsroom/PressAnnouncements/ucm517478.htm.

[27] Carey D, Zitomer D, Hristova K, Kappell A, McNamara P. Triclocarban Influences Antibiotic Resistance and Alters Anaerobic Digester Microbial Community Structure. *Environmental Science & Technology*. 2016;50(1), 126–134. 10.1021/acs.est.5b03080 26588246

[28] Kennedy RCM, Menn FM, Healy L, Fecteau KA, Hu P, Bae J, et al Early Life Triclocarban Exposure During Lactation Affects Neonate Rat Survival. *Reproductive Sciences*. 2015;22(1), 75–89. 10.1177/1933719114532844 10.1177/1933719114532844 24803507 PMC4527418

[29] Kennedy R, Fling R, Robeson M, Saxton A, Donnell R, Darcy J, et al Temporal Development of Gut Microbiota in Triclocarban Exposed Pregnant and Neonatal Rats. *Scientific Reports*. 2016;6, 33430 10.1038/srep33430 27646684 PMC5028839

[30] Syed A, Ghosh S, Love N, & Boles B. Triclosan Promotes Staphylococcus aureus Nasal Colonization. *Mbio*. 2014;5(2), e01015-13–e01015-13. 10.1128/mBio.01015-13 24713325 PMC3993860

[31] Yee A & Gilbert J Is triclosan harming your microbiome?. *Science*. 2016;353(6297), 348–349. 10.1126/science.aag2698 27463658

[32] Yueh M & Tukey R. Triclosan: A Widespread Environmental Toxicant with Many Biological Effects. *Annu*. *Rev*. *Pharmacol*. *Toxicol*. 2016;56(1), 251–272. 10.1146/annurev-pharmtox-010715-103417 26738475 PMC4774862

[33] Corp Wings. Santex Medicated Soap. 2016 Available from: http://www.wingscorp.com/content/exports/product_detail.php?h=1&b=3&c=7&l=1&m=219

[34] Lauber CL, Zhou N, Gordon JI, Knight R, Fierer N. Effect of storage conditions on the assessment of bacterial community structure in soil and human-associated samples. *FEMS Microbiology Letters*. 2010;307(1), 80–86. 10.1111/j.1574-6968.2010.01965.x 10.1111/j.1574-6968.2010.01965.x 20412303 PMC3148093

[35] Lozupone C, Lladser ME, Knights D, Stombaugh J, Knight R. UniFrac: an effective distance metric for microbial community comparison. *The ISME Journal*. 2011;5(2), 169–172. 10.1038/ismej.2010.133 10.1038/ismej.2010.133 20827291 PMC3105689

[36] R Core Team. R: A language and environment for statistical computing. R Foundation for Statistical Computing, Vienna, Austria 2015 Available from: https://www.R-project.org/.

[37] Segata N, Izard J, Waldron L, Gevers D, Miropolosky L, Garrett W, Huttenhower C. “Metagenomic biomarker discovery and explanation” *Genome Biology*. 2011;12:R60 10.1186/gb-2011-12-6-r60 21702898 PMC3218848

[38] Urban J, Fergus DJ, Savage AM, Ehlers M, Menninger HL, Dunn RR, et al The effect of habitual and experimental antiperspirant and deodorant product use on the armpit microbiome. *PeerJ*. 2016;4, e1605 10.7717/peerj.1605 10.7717/peerj.1605 26855863 PMC4741080

[39] Willems A. The Family *Comamonadaceae*. *The Prokaryotes*. 2014;pp. 777–851. 10.1007/978-3-642-30197-1_238

[40] WHO. *Antimicrobial resistance* 2017. Available at: http://www.who.int/mediacentre/factsheets/fs194/en/

[41] CDC *Antibiotic / Antimicrobial Resistance* 2017. Available at: https://www.cdc.gov/drugresistance/index.html

[42] Aiello A, Larson E, & Levy S. Consumer Antibacterial Soaps: Effective or Just Risky?. *Clinical Infectious Diseases*. 2007;45(Supplement 2), S137–S147. 10.1086/519255 17683018

[43] Giuliano C & Rybak M. Efficacy of Triclosan as an Antimicrobial Hand Soap and Its Potential Impact on Antimicrobial Resistance: A Focused Review. *Pharmacotherapy*: *The Journal Of Human Pharmacology And Drug Therapy*. 2015;35(3), 328–336. 10.1002/phar.1553 25809180

[44] Kim S, Moon H, Lee K, & Rhee M. Bactericidal effects of triclosan in soap both in vitro and in vivo. *Journal Of Antimicrobial Chemotherapy*. 2015;dkv275 10.1093/jac/dkv275 26374612

[45] Kim S & Rhee M. Microbicidal effects of plain soap vs triclocarban-based antibacterial soap. *Journal Of Hospital Infection*. 2016;94(3), 276–280. 10.1016/j.jhin.2016.07.010 27585555

[46] Johnson P, Koustas E, Vesterinen H, Sutton P, Atchley D, Kim A. et al Application of the Navigation Guide systematic review methodology to the evidence for developmental and reproductive toxicity of triclosan. *Environment International*. 2016;92–93, 716–728. 10.1016/j.envint.2016.03.009 27156197 PMC4951161

[47] Clemente JC, Pehrsson EC, Blaser MJ, Sandhu K, Gao Z, Wang B, et al "The microbiome of uncontacted Amerindians." Sci. Adv. 2015;1: 1–12. 10.1126/sciadv.1500183 PMC4517851 26229982

[48] Rook GAW and Brunet LR. Old friends for breakfast. Clinical & Experimental Allergy. 2005;35: 841–842. 10.1111/j.1365-2222.2005.02112.x 16008666

[49] Rook GAW, & Brunet LR. Microbes, immunoregulation, and the gut. *Gut*. 2005;54(3), 317–320. 10.1136/gut.2004.053785 10.1136/gut.2004.053785 15710972 PMC1774411

[50] Parker W & Ollerton J. Evolutionary biology and anthropology suggest biome reconstitution as a necessary approach toward dealing with immune disorders. *Evolution*, *Medicine*, *and Public Health*. 2013;(1), 89–103. 10.1093/emph/eot008 PMC3868394 24481190

