# Supplementary material for: Antibacterial soap use impacts skin microbial communities in rural Madagascar
Source: PLoS One. 2018 Aug 20;13(8):e0199899. doi: 10.1371/journal.pone.0199899 (PMC6101359; doi:10.1371/journal.pone.0199899)
Supplement: S2 Text — (DOCX) [file pone.0199899.s002.docx]

S2 – 16S metagenomic libarary prep guide

*Preparing 16S Ribosomal RNA Gene Amplicons for the*

*Illumina MiSeq System*

*Workflow Summary:*

1. Order amplicon primers–The protocol includes the primer pair sequences for the V3 and V4 region that create a single amplicon of approximately ~460 bp. The protocol also includes overhang adapter sequences that must be appended to the primer pair sequences for compatibility with Illumina index and sequencing adapters. Illumina does

not sell these primers. They must be ordered from a third party. See Amplicon Primers, on page 3 for more information on amplicon primers.

2. Prepare library–The protocol describes the steps to amplify the V3 and V4 region and using a limited cycle PCR, add Illumina sequencing adapters and dual‐index barcodes to the amplicon target. Using the full complement of Nextera XT indices, up to 96 libraries can be pooled together for sequencing.

3. Sequence on MiSeq–Using paired 300‐bp reads, and MiSeq v3 reagents, the ends of each read are overlapped to generate high‐quality, full‐length reads of the V3 and V4 region in a single 65‐hour run. The MiSeq run output is approximately > 20 million reads and, assuming 96 indexed samples, can generate > 100,000 reads per sample, commonly recognized as sufficient for metagenomic surveys.

4. Analyze on MSR or BaseSpace–The Metagenomics workflow is a secondary analysis option built into the MiSeq Reporter (on‐system software) or available on BaseSpace (cloud‐based software). The Metagenomics Workflow performs a taxonomic classification using the Greengenes database showing genus or species level classification in a graphical format.

This protocol can be used to sequence alternative regions of the 16S rRNA gene and for other targeted amplicon sequences of interest. When using this protocol for amplicon sequencing other than 16S rRNA, use the Generate FASTQ Workflow (secondary analysis option). For more information, see MiSeq Reporter Metagenomics Workflow, on page 20. DNA. A subsequent limited‐cycle amplification step is performed to add multiplexing indices and Illumina sequencing adapters. Libraries are normalized and pooled, and sequenced on the MiSeq system using v3 reagents.

*Amplicon Primers*

The gene‐specific sequences used in this protocol target the 16S V3 and V4 region. They are selected from the Klindworth et al. publication (Klindworth A, Pruesse E, Schweer T, Peplles J, Quast C, et al. (2013) Evaluation of general 16S ribosomal RNA gene PCR primers for classical and next‐generation sequencing‐based diversity studies. Nucleic Acids Res 41(1).) as the most promising bacterial primer pair. Illumina adapter overhang nucleotide sequences are added to the gene‐specific sequences. The full length primer sequences, using standard IUPAC nucleotide nomenclature, to follow the protocol targeting this region are:

16S Amplicon PCR Forward Primer = 5'

TCGTCGGCAGCGTCAGATGTGTATAAGAGACAGCCTACGGGNGGCWGCAG

16S Amplicon PCR Reverse Primer = 5'

GTCTCGTGGGCTCGGAGATGTGTATAAGAGACAGGACTACHVGGGTATCTAATCC

This method can also be utilized to target other regions on the genome (either for 16S with other sets of primer pairs, or non‐16S regions throughout the genome; ie any amplicon). The overhang adapter sequence must be added to the locus‐specific primer for the region to be targeted (Figure 1). The Illumina overhang adapter sequences to be added to locus‐specific sequences are:

Forward overhang: 5’ TCGTCGGCAGCGTCAGATGTGTATAAGAGACAG‐[locus specific sequence]

Reverse overhang: 5’ GTCTCGTGGGCTCGGAGATGTGTATAAGAGACAG‐[locus specific sequence]

The following considerations are recommended for designing other locus‐specific primers:

a) Illumina recommends targeting regions that result in an amplicon that when sequenced with paired‐end reads has at least ~50 bp of overlapping sequence in the middle. For example, if running 2x300 bp paired‐end reads Illumina recommends having an insert size of 550 bp or smaller so that the bases sequenced at the end of each read overlap.

b) The locus‐specific portion of primer (not including overhang sequence) must have a melting temperature (Tm) of 60°–65°C. You can use online PCR primer sequence analysis tools (e.g. http://www.idtdna.com/analyzer/Applications/OligoAnalyzer/) to

check the properties of primer designs. For the Tm calculation only, the gene‐specific portion must be used in calculation. For hairpin and dimer calculations, the fully assembled primer sequence (including the overhang) should be used.

c) Illumina recommends using standard desalting purification when ordering oligo primer sets.

*Amplicon PCR*

This step uses PCR to amplify template out of a DNA sample using region of interestspecific primers with overhang adapters attached. For more information on primer sequences, see Amplicon Primers, on page 3.

*Consumables*

| **Item** | **Quantity** | **Storage** |
| --- | --- | --- |
| Microbial Genomic DNA (5 ng/μl in 10 mM Tris pH 8.5) | 2.5 μl per sample | ‐15° to ‐25°C |
| Amplicon PCR Reverse Primer (1 μM) | 5 μl per sample | ‐15° to ‐25°C |
| Amplicon PCR Forward Primer (1 μM) | 5 μl per sample | ‐15° to ‐25°C |
| 2x KAPA HiFi HotStart ReadyMix | 12.5 μl per sample | ‐15° to ‐25°C |
| Microseal 'A' film |  |  |
| 96‐well 0.2 ml PCR plate | 1 plate |  |
| [Optional] Bioanalyzer chip (Agilent DNA 1000  kit catalog # 5067‐1504) |  |  |

*Procedure*

1. Set up the following reaction of DNA, 2x KAPA HiFi HotStart ReadyMix, and primers:

Volume

Microbial DNA (5 ng/μl) 2.5 μl

Amplicon PCR Forward Primer 1 μM 5 μl

Amplicon PCR Reverse Primer 1 μM 5 μl

2x KAPA HiFi HotStart ReadyMix 12.5 μl

Total 25 μl

2. Seal plate and perform PCR in a thermal cycler using the following program:

• 95°C for 3 minutes

• 25 cycles of:

— 95°C for 30 seconds

— 55°C for 30 seconds

— 72°C for 30 seconds

• 72°C for 5 minutes

• Hold at 4°C

3 [Optional] Run 1 μl of the PCR product on a Bioanalyzer DNA 1000 chip to verify the size. Using the V3 and V4 primer pairs in the protocol, the expected size on a Bioanalyzer trace after the Amplicon PCR step is ~550 bp.

*PCR Clean‐Up*

This step uses AMPure XP beads to purify the 16S V3 and V4 amplicon away from free primers and primer dimer species.

*Consumables*

| **Item** | **Quantity** | **Storage** |
| --- | --- | --- |
| 10 mM Tris pH 8.5 | 52.5 μl per sample | ‐15° to ‐25°C |
| AMPure XP beads | 20 μl per sample | 2° to 8°C |
| Freshly Prepared 80% Ethanol (EtOH) | 400 μl per sample |  |
| 96‐well 0.2 ml PCR plate | 1 plate |  |
| [Optional] Microseal 'B' film |  |  |
| [Optional] 96‐well MIDI plate | 1 plate |  |

*Preparation*

Bring the AMPure XP beads to room temperature.

*Procedure*

1. Centrifuge the Amplicon PCR plate at 1,000 × g at 20°C for 1 minute to collect

condensation, carefully remove seal.

2. [Optional - for use with shaker for mixing] Using a multichannel pipette set to 25 μl, transfer the entire Amplicon PCR product from the PCR plate to the MIDI plate. Change tips between samples.

3. Vortex the AMPure XP beads for 30 seconds to make sure that the beads are evenly dispersed. Add an appropriate volume of beads to a trough depending on the number of samples processing.

4. Using a multichannel pipette, add 20 μl of AMPure XP beads to each well of the Amplicon PCR plate. Change tips between columns.

5. Gently pipette entire volume up and down 10 times if using a 96‐well PCR plate or seal plate and shake at 1800 rpm for 2 minutes if using a MIDI plate.

6. Incubate at room temperature without shaking for 5 minutes.

7. Place the plate on a magnetic stand for 2 minutes or until the supernatant has cleared.

8. With the Amplicon PCR plate on the magnetic stand, use a multichannel pipette to remove and discard the supernatant. Change tips between samples.

9. With the Amplicon PCR plate on the magnetic stand, wash the beads with freshly prepared 80% ethanol as follows:

a) Using a multichannel pipette, add 200 μl of freshly prepared 80% ethanol to each sample well.

b) Incubate the plate on the magnetic stand for 30 seconds.

c) Carefully remove and discard the supernatant.

10. With the Amplicon PCR plate on the magnetic stand, perform a second ethanol wash as follows:

a) Using a multichannel pipette, add 200 μl of freshly prepared 80% ethanol to each sample well.

b) Incubate the plate on the magnetic stand for 30 seconds.

c) Carefully remove and discard the supernatant.

d )Use a P20 multichannel pipette with fine pipette tips to remove excess ethanol.

11. With the Amplicon PCR plate still on the magnetic stand, allow the beads to air‐dry for 10 minutes.

12. Remove the Amplicon PCR plate from the magnetic stand. Using a multichannel pipette, add 52.5 μl of 10 mM Tris pH 8.5 to each well of the Amplicon PCR plate.

13. Gently pipette mix up and down 10 times, changing tips after each column (or seal plate and shake at 1800 rpm for 2 minutes). Make sure that beads are fully resuspended.

14. Incubate at room temperature for 2 minutes.

15. Place the plate on the magnetic stand for 2 minutes or until the supernatant has cleared.

16. Using a multichannel pipette, carefully transfer 50 μl of the supernatant from the Amplicon PCR plate to a new 96‐well PCR plate. Change tips between samples to avoid cross‐contamination.

*Index PCR*

This step attaches dual indices and Illumina sequencing adapters using the Nextera XT Index Kit.

*Consumables*

| **Item** | **Quantity** | **Storage** |
| --- | --- | --- |
| 2x KAPA HiFi HotStart ReadyMix | 25 μl per sample | ‐15° to ‐25°C |
| Nextera XT Index 1 Primers (N7XX) from the Nextera XT Index kit (FC‐131‐1001 or FC‐131‐1002) | 5 μl per sample | ‐15° to ‐25°C |
| Nextera XT Index 2 Primers (S5XX) from the Nextera XT Index kit (FC‐131‐1001 or FC‐131‐1002) | 5 μl per sample | ‐15° to ‐25°C |
| PCR Grade Water | 10 μl per sample |  |
| TruSeq Index Plate Fixture (FC‐130‐1005) | 1 |  |
| 96‐well 0.2 ml PCR plate | 1 plate |  |
| Microseal 'A' film | 1 |  |

*Procedure*

1. Using a multichannel pipette, transfer 5 μl from each well to a new 96‐well plate. The remaining 45 μl is not used in the protocol and can be stored for other uses.

2. Arrange the Index 1 and 2 primers in a rack (i.e. the TruSeq Index Plate Fixture) using the following arrangements as needed:

a) Arrange Index 2 primer tubes (white caps, clear solution) vertically, aligned with rows A through H.

b) Arrange Index 1 primer tubes (orange caps, yellow solution) horizontally, aligned with columns 1 through 12.

3. Place the 96‐well PCR plate with the 5 μl of resuspended PCR product DNA in the TruSeq Index Plate Fixture.

4. Set up the following reaction of DNA, Index 1 and 2 primers, 2x KAPA HiFi HotStart ReadyMix, and PCR Grade water:

Volume

DNA 5 μl

Nextera XT Index Primer 1 (N7xx) 5 μl

Nextera XT Index Primer 2 (S5xx) 5 μl

2x KAPA HiFi HotStart ReadyMix 25 μl

PCR Grade water 10 μl

Total 50 μl

5. Gently pipette up and down 10 times to mix.

6. Cover the plate with Microseal 'A'.

7. Centrifuge the plate at 1,000 × g at 20°C for 1 minute.

8. Perform PCR on a thermal cycler using the following program:

• 95°C for 3 minutes

• 8 cycles of:

— 95°C for 30 seconds

— 55°C for 30 seconds

— 72°C for 30 seconds

• 72°C for 5 minutes

• Hold at 4°C

*PCR Clean‐Up 2*

This step uses AMPure XP beads to clean up the final library before quantification.

*Consumables*

| **Item** | **Quantity** | **Storage** |
| --- | --- | --- |
| 10 mM Tris pH 8.5 | 27.5 μl per sample | ‐15° to ‐25°C |
| AMPure XP beads | 56 μl per sample | 2° to 8°C |
| Freshly Prepared 80% Ethanol (EtOH) | 400 μl per sample |  |
| 96‐well 0.2 ml PCR plate | 1 plate |  |
| [Optional] Microseal 'B' film |  |  |
| [Optional] 96‐well MIDI plate | 1 plate |  |

*Procedure*

1. Centrifuge the Index PCR plate at 280 × g at 20°C for 1 minute to collect condensation.

2. [Optional - for use with shaker for mixing] Using a multichannel pipette set to 50 μl, transfer the entire Index PCR product from the PCR plate to the MIDI plate. Change tips between samples.

3. Vortex the AMPure XP beads for 30 seconds to make sure that the beads are evenly dispersed. Add an appropriate volume of beads to a trough.

4. Using a multichannel pipette, add 56 μl of AMPure XP beads to each well of the Index PCR plate.

5. Gently pipette mix up and down 10 times if using a 96‐well PCR plate or seal plate and shake at 1800 rpm for 2 minutes if using a MIDI plate.

6. Incubate at room temperature without shaking for 5 minutes.

7. Place the plate on a magnetic stand for 2 minutes or until the supernatant has cleared.

8. With the Index PCR plate on the magnetic stand, use a multichannel pipette to remove and discard the supernatant. Change tips between samples.

9. With the Index PCR plate on the magnetic stand, wash the beads with freshly prepared 80% ethanol as follows:

a) Using a multichannel pipette, add 200 μl of freshly prepared 80% ethanol to each

sample well.

b) Incubate the plate on the magnetic stand for 30 seconds.

c) Carefully remove and discard the supernatant.

10. With the Index PCR plate on the magnetic stand, perform a second ethanol wash as follows:

a) Using a multichannel pipette, add 200 μl of freshly prepared 80% ethanol to each

sample well.

b) Incubate the plate on the magnetic stand for 30 seconds.

c) Carefully remove and discard the supernatant.

d) Use a P20 multichannel pipette with fine pipette tips to remove excess ethanol.

11. With the Index PCR plate still on the magnetic stand, allow the beads to air‐dry for 10 minutes.

12. Remove the Index PCR plate from the magnetic stand. Using a multichannel pipette, add 27.5 μl of 10 mM Tris pH 8.5 to each well of the Index PCR plate.

13. If using a 96‐well PCR plate, gently pipette mix up and down 10 times until beads are fully resuspended, changing tips after each column. If using a MIDI plate, seal plate and shake at 1800 rpm for 2 minutes.

14. Incubate at room temperature for 2 minutes.

15. Place the plate on the magnetic stand for 2 minutes or until the supernatant has cleared.

16. Using a multichannel pipette, carefully transfer 25 μl of the supernatant from the Index PCR plate to a new 96‐well PCR plate. Change tips between samples to avoid cross contamination.

*Library Quantification, Normalization, and Pooling*

Illumina recommends quantifying your libraries using a fluorometric quantification method that uses dsDNA binding dyes. Calculate DNA concentration in nM, based on the size of DNA amplicons as determined by an Agilent Technologies 2100 Bioanalyzer trace: (concentration in ng/μl) / (660 g/mol × average library size) × 106 = concentration in nM

For example:

15 ng/μl / (660 g/mol × 500) × 106 = 45 nM

Dilute concentrated final library using Resuspension Buffer (RSB) or 10 mM Tris pH 8.5 to 4 nM. Aliquot 5 μl of diluted DNA from each library and mix aliquots for pooling libraries with unique indices. Depending on coverage needs, up to 96 libraries can be pooled for one MiSeq run.

For metagenomics samples, >100,000 reads per sample is sufficient to fully survey the bacterial composition. This number of reads allows for sample pooling to the maximum level of 96 libraries, given the MiSeq output of > 20 million reads.

*Library Denaturing and MiSeq Sample Loading*

In preparation for cluster generation and sequencing, pooled libraries are denatured with NaOH, diluted with hybridization buffer, and then heat denatured before MiSeq sequencing. Each run must include a minimum of 5% PhiX to serve as an internal control for these lowdiversity libraries. Illumina recommends using MiSeq v3 reagent kits for improved run metrics.

*Consumables*

| **Item** | **Quantity** | **Storage** |
| --- | --- | --- |
| 10 mM Tris pH 8.5 or RSB (Resuspension Buffer) | 6 μl | ‐15° to ‐25°C |
| HT1 (Hybridization Buffer) | 1540 μl | ‐15° to ‐25°C |
| 0.2 N NaOH (less than a week old) | 10 μl | ‐15° to ‐25°C |
| PhiX Control Kit v3 (FC‐110‐3001) | 4 μl | ‐15° to ‐25°C |
| MiSeq reagent cartridge | 1 cartridge | ‐15° to ‐25°C |
| 1.7 ml microcentrifuge tubes | 3 tubes |  |
| 2.5 L ice bucket |  |  |

*Preparation*

1. Set a heat block suitable for 1.7 ml microcentrifuge tubes to 96°C

2. Remove a MiSeq reagent cartridge from ‐15°C to ‐25°C storage and thaw at room temperature.

3. In an ice bucket, prepare an ice‐water bath by combining 3 parts ice and 1 part water.

*Denature DNA*

1. Combine the following volumes of pooled final DNA library and freshly diluted 0.2 N

NaOH in a microcentrifuge tube:

• 4 nM pooled library (5 μl)

• 0.2 N NaOH (5 μl)

2. Set aside the remaining dilution of 0.2 N NaOH to prepare a PhiX control within the next 12 hours.

3. Vortex briefly to mix the sample solution, and then centrifuge the sample solution at 280 × g at 20°C for 1 minute.

4. Incubate for 5 minutes at room temperature to denature the DNA into single strands.

5. Add the following volume of pre‐chilled HT1 to the tube containing denatured DNA:

• Denatured DNA (10 μl)

• Pre‐chilled HT1 (990 μl)

Adding the HT1 results in a 20 pM denatured library in 1 mM NaOH.

6. Place the denatured DNA on ice until you are ready to proceed to final dilution.

*Dilute Denatured DNA*

1. Dilute the denatured DNA to the desired concentration using the following example:

Final Concentration 2 pM 4 pM 6 pM 8 pM 10 pM

20 pM denatured library 60 μl 120 μl 180 μl 240 μl 300 μl

Pre‐chilled HT1 540 μl 480 μl 420 μl 360 μl 300 μl

2. Invert several times to mix and then pulse centrifuge the DNA solution.

3. Place the denatured and diluted DNA on ice.

*Denature and Dilution of PhiX Control*

Use the following instructions to denature and dilute the 10 nM PhiX library to the same loading concentration as the Amplicon library. The final library mixture must contain at least 5% PhiX.

1. Combine the following volumes to dilute the PhiX library to 4 nM:

• 10 nM PhiX library (2 μl)

• 10 mM Tris pH 8.5 (3 μl)

2. Combine the following volumes of 4 nM PhiX and 0.2 N NaOH in a microcentrifuge tube:

• 4 nM PhiX library (5 μl)

• 0.2 N NaOH (5 μl)

3. Vortex briefly to mix the 2 nM PhiX library solution.

4. Incubate for 5 minutes at room temperature to denature the PhiX library into single strands.

5. Add the following volumes of pre‐chilled HT1 to the tube containing denatured PhiX library to result in a 20 pM PhiX library:

• Denatured PhiX library (10 μl)

• Pre‐chilled HT1 (990 μl)

6. Dilute the denatured 20 pM PhiX library to the same loading concentration as the Amplicon library as follows:

Final Concentration 2 pM 4 pM 6 pM 8 pM 10 pM

20 pM denatured library 60 μl 120 μl 180 μl 240 μl 300 μl

Pre‐chilled HT1 540 μl 480 μl 420 μl 360 μl 300 μl

7. Invert several times to mix and then pulse centrifuge the DNA solution.

8. Place the denatured and diluted PhiX on ice.

*Combine Amplicon Library and PhiX Control*

1. Combine the following volumes of denatured PhiX control library and your denatured amplicon library in a microcentrifuge tube:

• Denatured and diluted PhiX control (30 μl)

• Denatured and diluted amplicon library (570 μl)

2. Set the combined sample library and PhiX control aside on ice until you are ready to heat denature the mixture immediately before loading it onto the MiSeq v3 reagent cartridge.

3. Using a heat block, incubate the combined library and PhiX control tube at 96°C for 2 minutes.

4. After the incubation, invert the tube 1–2 times to mix and immediately place in the ice water bath.

5. Keep the tube in the ice‐water bath for 5 minutes.
